# Evaluation of the Bioactive Potential of Secondary Metabolites Produced by a New Marine *Micrococcus* Species Isolated from the Persian Gulf

**Published:** 2020

**Authors:** Hamid Reza Karbalaei-Heidari, Mozhdeh Partovifar, Mina Memarpoor-Yazdi

**Affiliations:** 1. Molecular Biotechnology Lab., Department of Biology, Faculty of Science, Shiraz University, Shiraz, Iran; 2. Institute of Biotechnology, Shiraz University, Shiraz, Iran

**Keywords:** Antibacterial effect, Antioxidant activity, Bioactive compounds, Marine bacteria, Persian Gulf

## Abstract

**Background:**

In the present work, a newly isolated marine bacterium, *Micrococcus* sp. MP76, from coastal area of Persian Gulf around Bushehr province, Iran, was identified with the ability to produce bioactive compounds.

**Methods:**

The pigment production was optimized by changing carbon and nitrogen sources in bacterial growth media at 28°*C* and 220 *rpm* for 5 days. Partial purification of the pigment was carried out using suitable solvents.

**Results:**

Maximum pigment extract was achieved (1.4 *g/l*) when cultured in the medi*um* containing 0.5% (v/v) molasses, 0.5% (w/v) peptone, 1% (w/v) sea salt, 0.01% (w/v) potassium phosphate, and 0.05% (w/v) yeast extract, pH=7.0. Antibacterial effect assessment of the extract against pathogenic bacteria revealed the MIC values in the range of 4.2–7.5 *mg/ml* depending on different pathogens. The pigment extracted from medium supplemented by molasses and ammonium sulfate had 81% radical scavenging activity, and its IC_50_ value was 0.28 *mg/ml*.

**Conclusion:**

The newly isolated strain of *Micrococcus* genus from the Persian Gulf revealed a valuable source to access worth medicinal ingredients when cultured under optimized conditions.

## Introduction

Marine bacteria are invaluable resources in providing bioactive compounds 1,2. Marine natural products have significant roles in biomedical research and pharmaceutical industry. In the last decades, a number of bioactive compounds from marine micro-organisms have been identified 3–6. Although many of these compounds have been used as pharmaceutics, screening of bioactive agents to find the new chemical structures is still ongoing. The bioactive compounds are typically synthesized during end of the stationary phase of microorganism life cycle. Some of these compounds have been introduced as antibiotics, which play critical roles in surviving and thriving of micro-organisms in bacterial populations or to resist against nutritional stresses 7,8. On the other hand, pigments are one of the most important secondary metabolites which are widely produced by micro-organisms. They are non-toxic, non-carcinogenic and biocompatible and have been welcomed as safe pharmaceuticals. The immune-modulation, antioxidant and anti-carcinogenic properties of carotenoid compounds, as the most important identified pigments, have been reported as well 9,10.

Actinobacteria are a class of gram-positive bacteria which are well known as pioneers in the production of a wide variety of useful secondary metabolites such as antitumor agents, antibiotics, antioxidants, pigments and enzymes 11,12. Among actinobacterial genera, *Micrococcus* has been reported to contain significant potential to produce a large number of useful compounds. For instance, canthaxanthin (4′,4′-diketo-13-carotene) and α- and β-carotene derivatives were purified and characterized from *Micrococcus roseus (M. roseus)* 13, 14. Moreover, crude pigments from *Micrococcus luteus (M. luteus)* revealed antibacterial effects against *Staphylococcus* sp., *Klebsiella* sp., and *Pseudomonas* sp. 15.

According to this background, the aims of the current study can be summarized as isolation and characterization of a pigment producing bacterium from Persian Gulf, optimization of pigment production using different carbon and nitrogen sources, and finally evaluation of its antibacterial and antioxidant potentials.

## Materials and Methods

### Isolation of pigment producing bacteria

Pigment producing bacteria were isolated from water samples collected from coastal area of Persian Gulf around Bushehr province, Iran. Micro-organisms were precultured on nutrient agar plates (Difco) containing 3% (w/v) sea salt at 30°*C*, pH=8.0 for 5 days. Primary culture medium was composed of 0.3% (w/v) yeast extract, 3% (w/v) sea salt and 0.5% (w/v) casein hydrolysates (pH∼8.0). The effect of different carbon and nitrogen sources (0.5%) on pigment production yield was investigated. To produce secondary metabolites, 1% (v/v) seed culture of the strain MP76 was inoculated into production medium and incubated at 28°*C* and 220 *rpm* for 5 days.

### Biochemical and molecular characterization of the strain MP76

Morphological and molecular features of the isolate (MP76) were characterized. Biochemical tests were carried out according to the prevalent procedures described by Quinn *et al* 16. For phylogenetic classification, the 16S rRNA gene sequence of the strain MP76 was amplified with forward primer of HRK1 (5′-AC-TCCTACGGGAGGCAGCAG-3′), and reverse primer of HRK2 (5′-TGACGGGCGGTGTGTACAAG-3′) us-ing a thermocycler (Biomerta, USA). After nucleotide sequencing, alignment analysis and phylogenetic tree construction were carried out by MEGA 6.06 software 17.

### Optimization of bacterial growth and pigment production

To investigate the effect of pH and sea salt concentration on the strain MP76 growth, biomass dry weight was measured in production media containing 1–10% w/v sea salt and the pH range of 5.0–9.0. To prepare the extract from the bacterial cells, biomass collected from 5-day culture was suspended in acidic methanol (methanol containing 4% v/v HCl 1N), vortexed gently for 5 min, then separated by centrifugation at 8000 x g for 10 *min*. The obtained crude pigment extract was dried at 40°*C* and used for further analysis.

### Study on the pigment extract bioactivities

***Antibacterial effect:*** Antibacterial potential of the pigment extract was investigated by Well Diffusion method (WD), as described by Valgas *et al* 18, with a slight modification. Antibacterial effects of the pigment extract were studied against the pathogenic bacteria such as *Pseudomonas aeruginosa (P. aeruginosa)* (ATCC 27853), *Escherichia coli (E. coli)* (ATCC 25922) and *Staphylococcus aureus* (*S*. *aureus)* (ATCC 25923). The discs were impregnated with 20 *μl* of the pigment extract (6.3 *mg/ml*) and placed on the agar plates. After 16–18 *hr* incubation at 37°*C*, diameter of the clear zone of growth inhibition was measured.

Minimum Inhibitory Concentration (MIC) is defined as the lowest concentration of antibacterial compound which prevents visible growth of a bacterium after 24 *hr* of incubation at 37°*C*.

Three pathogenic bacteria were cultivated in NB for 18 *hr* at 37°*C*, then MTT solution (10 *μl*, 0.5 *mg/ml*) was added into each well and incubated at 37°*C* for 4 *hr*. After adding 100 *μl* of the solubilizing buffer and incubation in the dark for 24 *hr*, the absorbance at 590 and 630 *nm* was recorded using an ELISA reader. All experiments were carried out in triplicate.

### Antioxidant assay

DPPH scavenging activity was determined as described by Memarpoor-Yazdi *et al* 19, with slight modification. Briefly, 660 *μl* of 0.1 *mM* DPPH was mixed with 40 *μl* of the pigment extract in a final concentration of 0.4 *mg/ml*. After mixing vigorously for 2 *min*, the mixture was kept at 37°*C* for 30 *min*. Then, the absorbance of the mixture was recorded at 517 *nm* using a UV-Vis spectrophotometer (Shimadzu, UV. 120-02). DPPH Radical Scavenging Activity (RSA) was determined based on the following equation:

$$\text{RSA}\;(\%)=[({\text{A}}_{\text{control}}-{\text{A}}_{\text{sample}})/{\text{A}}_{\text{control}}]\times 100$$

Trolox is commonly employed as a standard or positive control in antioxidant assays.

## Results and Discussion

### Isolation and characterization of the pigment producing bacterium

**Figure 1.**
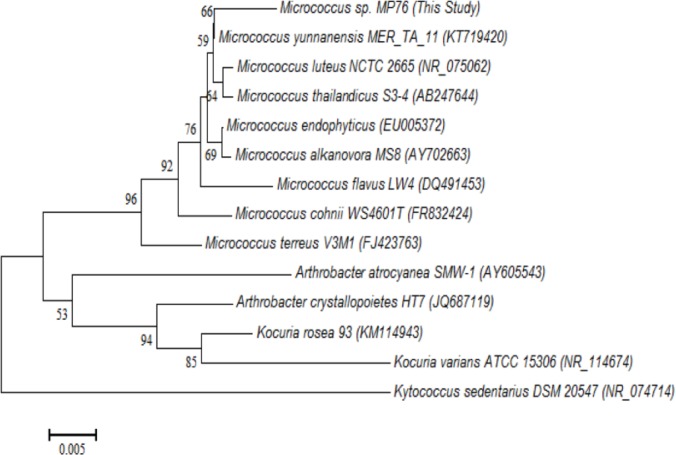
Phylogenetic tree of *Micrococcus* sp. MP76.

Screening and production of bioactive compounds and finding the economical culture medium has gained much attraction among researchers. Accordingly, in this study, Persian Gulf coast area was studied to isolate and characterize an efficient bacterium. The strain MP76 was isolated from coastal area of Bushehr province and characterized as a pigment producing bacterium. Isolate MP76 was identified as a gram-positive coccus, with yellow color colonies. Some biochemical characteristics of the isolate (MP76) are determined and summarized in table 1. For the molecular characterization, a consensus sequence of 911 *bp* fragment of its 16S rDNA gene was identified and deposited in the GenBank database under accession number KT804695. The sequence alignment revealed that the isolate MP76 belongs to *Micrococcus*, a genus of bacteria in the Micrococcaceae family and named as *Micrococcus* sp. strain MP76. The phylogenetic tree (Figure 1) showed that *Micrococcus* sp. strain MP76 is closely related to *Micrococcus yannanensis* (KT719420), a gram-positive bacterium isolated from a plant 20.

**Table 1.** Biochemical characterization of the isolated strain MP76

**Test**	**Result**
Gram staining	Gram positive
Morphology	Circular
Pigment	Yellow
Indole production	−
Lysine dehydroxylase	+
H_2_S production	−
Urease	−
Oxidase	+
Catalase	+
Coagulase	+
Gelatinase	−
Sucrose/Glucose fermentation	−/−

### Optimization of bacterial growth and biomass production

**Figure 2.**
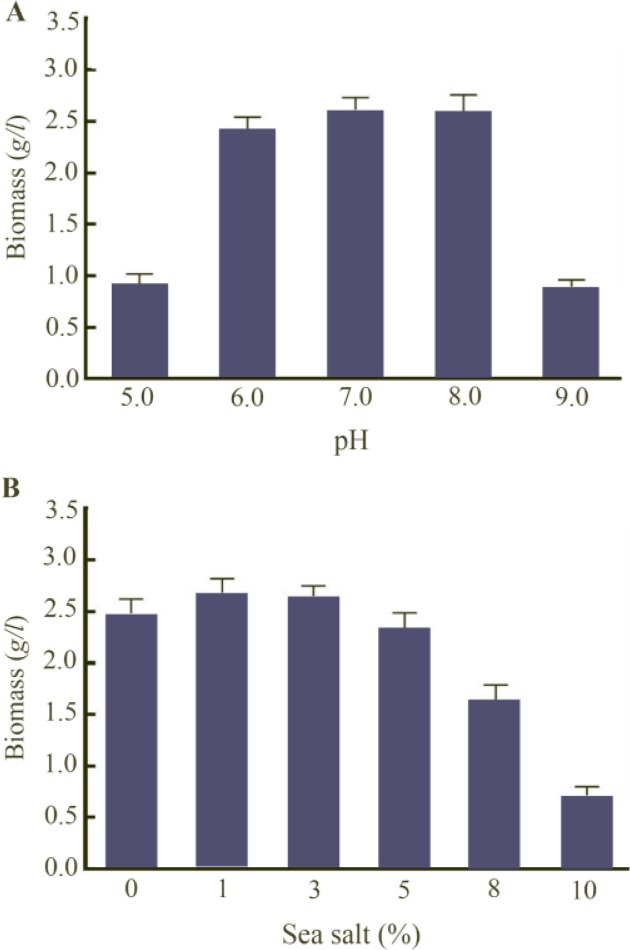
Effect of pH (A), and sea salt concentration (B) on biomass dry weight.

**Figure 3.**
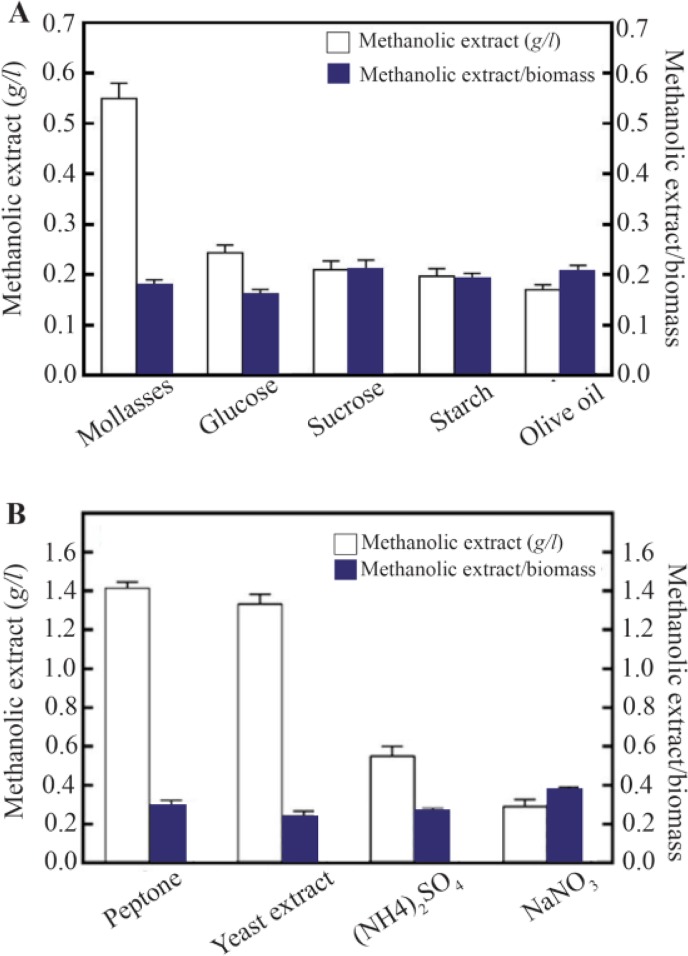
Effect of carbon (A), and nitrogen sources (B) on methanolic extract and the ratio of methanol extract to biomass.

The maximum biomass production was observed at the pH range of 6.0–8.0 and the highest was obtained to be 2.7 *g/l* at pH 7.0 (Figure 2A). Moreover, the noticeable reductions in biomass weight to about 0.95 and 0.8 *g/l* were observed at pH=5.0 and 9.0, respectively. The highest growth rate was obtained at 0–3% salt (2.8 *g/l* at 1% sea salt), although it was dropped significantly at higher than 5% (Figure 2B). Effect of different carbon and nitrogen sources revealed that maximum methanolic extract amount was obtained in molasses medium, which was 0.58 *g/l*, while it declined to 0.18, 0.2, 0.23 and 0.26 *g/l* in the medium containing olive oil, starch, sucrose and glucose, respectively (Figure 3A). However, the ratio of methanolic extract to biomass, defined as the pigment production yield, was relatively unchanged. As shown in figure 2B, the highest methanolic extract was 1.4 *g/l* in the medium supplemented by peptone. Although the methanolic extract for peptone and yeast extract were much higher than that of other nitrogen sources, no remarkable difference in pigment production yield was shown among the tested nitrogen sources (Figure 3B).

### Pigment characterization

*Micrococcus* sp. strain MP76 produced a yellow pigment, which was dissolved in methanolic extraction of the biomass. The absorption spectrum of chloroform extract of the pigment had three maximum wavelengths of 418, 437 and 448 *nm*, showing the most similarity with carotenoids. The maximum wavelengths of the obtained pigment were similar with carotenoid produced by *M. luteus* 15. It has been reported that *M. luteus*, isolated from sea water, was able to produce a yellow pigment with antibacterial activity against *Klebsiella*, *P. aeruginosa* and *S. aureus* 15.

### Determination of bioactivities of the extracted pigment

**Figure 4.**
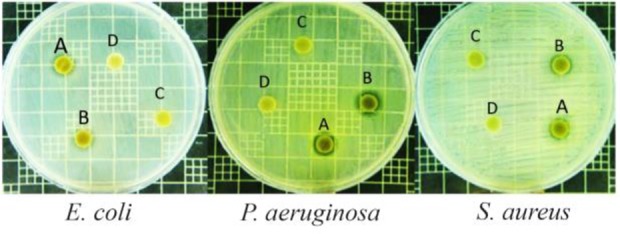
Antibacterial effects of pigment extract obtained from different nitrogen sources including peptone (A), yeast extract (B), (NH_4_)_2_ SO_4_ (C), and NaNO_3_ (D)

***Antibacterial activity:*** Primary antibacterial tests indicated that the pigment extracts derived only from culture containing yeast extract and peptone as nitrogen sources have shown positive results (Figure 4), while among various carbon sources, all pigment extracts had almost the similar inhibitory effects at the same concentrations against *E. coli, P. aeruginosa* and *S. ureus* (data not shown). Accordingly, the active pigment extracts were selected to determine IC_50_ and MIC values. The MIC values of the methanolic pigment extract were obtained to be 4.2, 5.0 and 7.5 *mg/ml* against *S. aureus*, *P. aeruginosa*, *E. coli*, respectively. Furthermore, the IC_50_ values against *S. aureus*, *P. aeruginosa*, *E. coli* were 3.4, 4.8, 4.8 *mg/ml*, respectively. The pigment extract revealed the highest antibacterial activity against the drug resistant hospital bacterium, *S. aureus.* It has been reported that ethyl acetate extract of the supernatant, produced by a species of *Micrococcus* isolated from Bamboo tree waste, showed the MIC value of 256 *μg/ml* against *S. aureus* 21. Moreover, Duraikannu *et al* reported almost similar antibacterial effect from the crude extract of a new marine soil isolate, *Streptomyces gancidicus* VITSD1 22.

**Figure S1.**
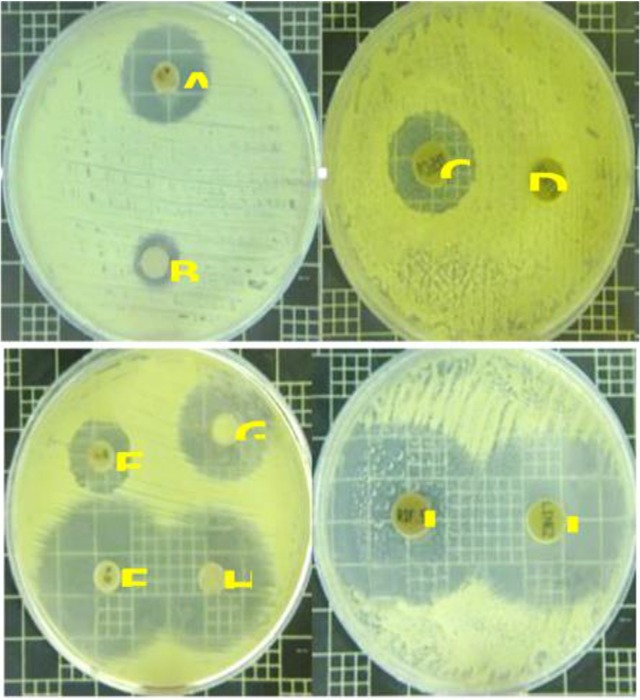
Susceptibility of *Micrococcus* sp. MP76 to different antibiotics including: Ciprofloxacin (A); Chloramphenicol (B); Daptomycin (C); Streptomycin (D); Vancomycin (E); Penicillin (F); Trimethoprim (G); Erythromycin (H); Rifampicin (I) and Linezolid (J).

Investigation of the bacterium susceptibility to different antibiotics revealed that *Micrococcus* sp. strain MP76 was susceptible to all tested antibiotics except for streptomycin (Figure S1) 23. Based on the observed resistance to streptomycin, it can be suggested that the antibiotic produced by *Micrococcus* sp. MP76 might belong to aminoglycoside family of antibiotics that includes streptomycin.

### Antioxidant activity

Ability of the pigment extracts obtained from different carbon and nitrogen sources for DPPH Radical Scavenging Activity (RSA) was determined at the concentration of 0.4 *mg/ml*. As illustrated in table 2, among carbon sources, media supplemented by molasses and olive oil showed the highest and the least RSA values, respectively. RSA value of molasses was 1.6-, 3.7- and 4.2-fold higher than that of sucrose, starch and olive oil, respectively. RSA value of ammonium sulfate was 1.8 and 1.2-fold more than that of sodium nitrate and yeast extract, respectively. The pigment extracted from media supplemented by molasses and ammonium sulfate had the highest RSA value (81%) and their IC_50_ value was 0.28 *mg/ml*. The most effective antioxidant activity of the pigment extract was obtained in molasses medium, which can be considered as a cost-effective and valuable source to obtain this bioactive compound from the strain MP76. Sugar beet molasses is composed of sucrose (near 48%) and some other compounds in small scales including nitrogen, sulfur and other minerals such as calcium, potassium, chloride and oxalate 24.

**Table 2.** DPPH scavenging capacity (%) of the obtained ethanol extract from different carbon and nitrogen sources

**Source**	**RSA (%)^a^**	**Trolox equivalent concentration(*mM*)**
**Carbon sources**		
Molasses	81.68	36.47
Glucose	51..39	22.35
Sucrose	50.29	21.76
Starch	22.00	8.82
Olive oil	19.50	7.64
**Nitrogen sources**		
Peptone	61.28	27.05
Yeas extract	66.11	28.82
(NH4)_2_ SO_4_	81.68	35.88
NaNO_3_	43.35	18.82

a) The average of standard deviations was ± 2%.

## Conclusion

Based on the data of this research, it can be concluded that untapped offshore resources such as the Persian Gulf have the potential to isolate bacteria with the ability to produce bioactive compounds under optimized conditions, and research to identify these bacteria as well as purification of active compounds can be a way to discover the effective antibacterial compounds.
